# Supplementary material for: Genomic analysis of Coccomyxa viridis, a common low-abundance alga associated with lichen symbioses
Source: Sci Rep. 2023 Dec 2;13:21285. doi: 10.1038/s41598-023-48637-w (PMC10693582; doi:10.1038/s41598-023-48637-w)
Supplement: Supplementary file 1 — Supplementary Figures. [file 41598_2023_48637_MOESM1_ESM.docx]

**Genomic analysis of *Coccomyxa viridis*, a common low-abundance alga associated with lichen symbioses**

Gulnara Tagirdzhanova^1^, Klara Scharnagl^1,2^, Xia Yan^1^ and Nicholas J. Talbot^1^

^1^The Sainsbury Laboratory, University of East Anglia, Norwich Research Park, Colney Lane, Norwich NR4 7UH, UK

^2^University & Jepson Herbaria, University of California Berkeley, Valley Life Sciences Building, Berkeley, CA 94720, USA


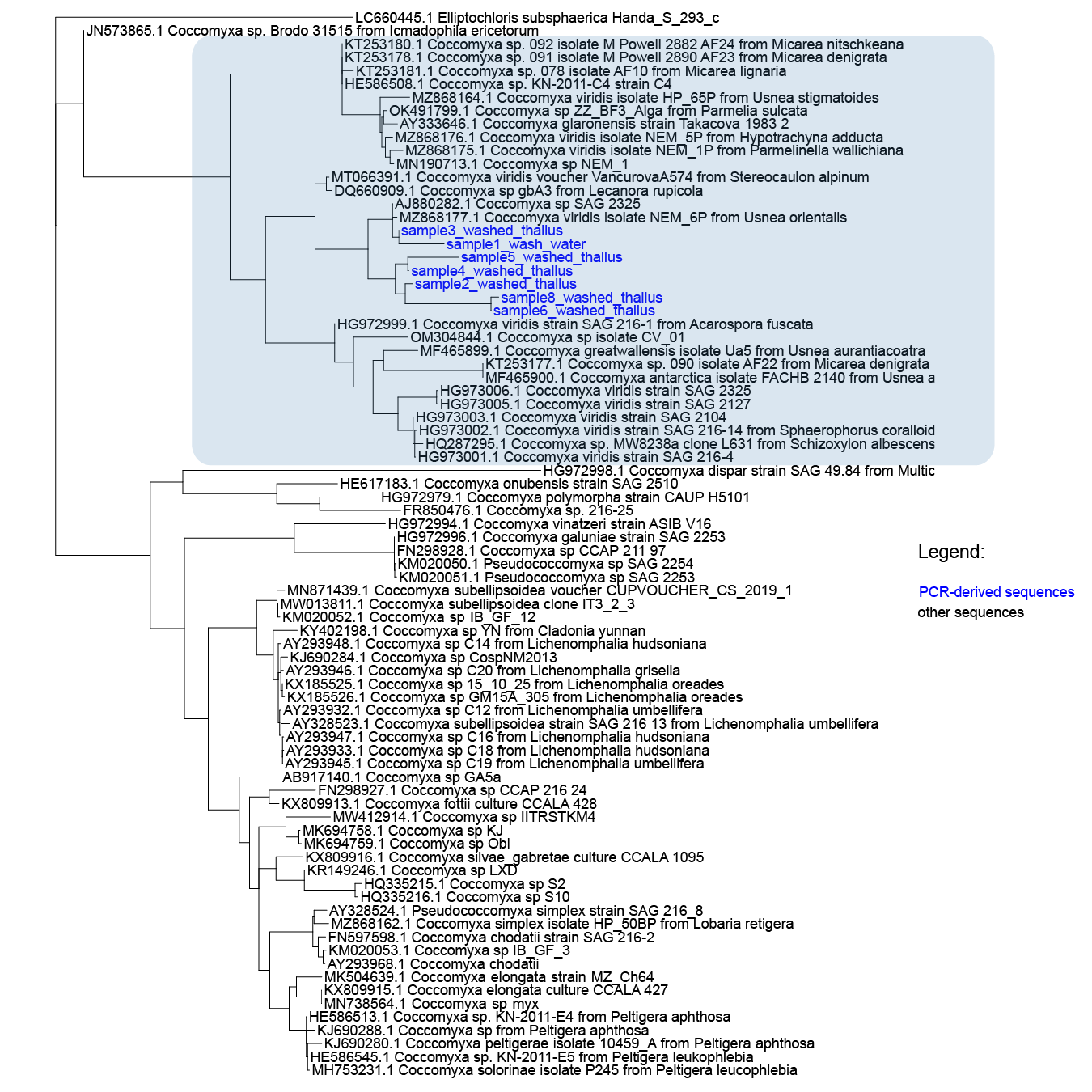


Fig. S1. Maximum-likelihood phylogenetic tree of *Coccomyxa* ITS. Sequences produced by PCR highlighted in blue font. The blue rectangle shows the *C. viridis* clade.


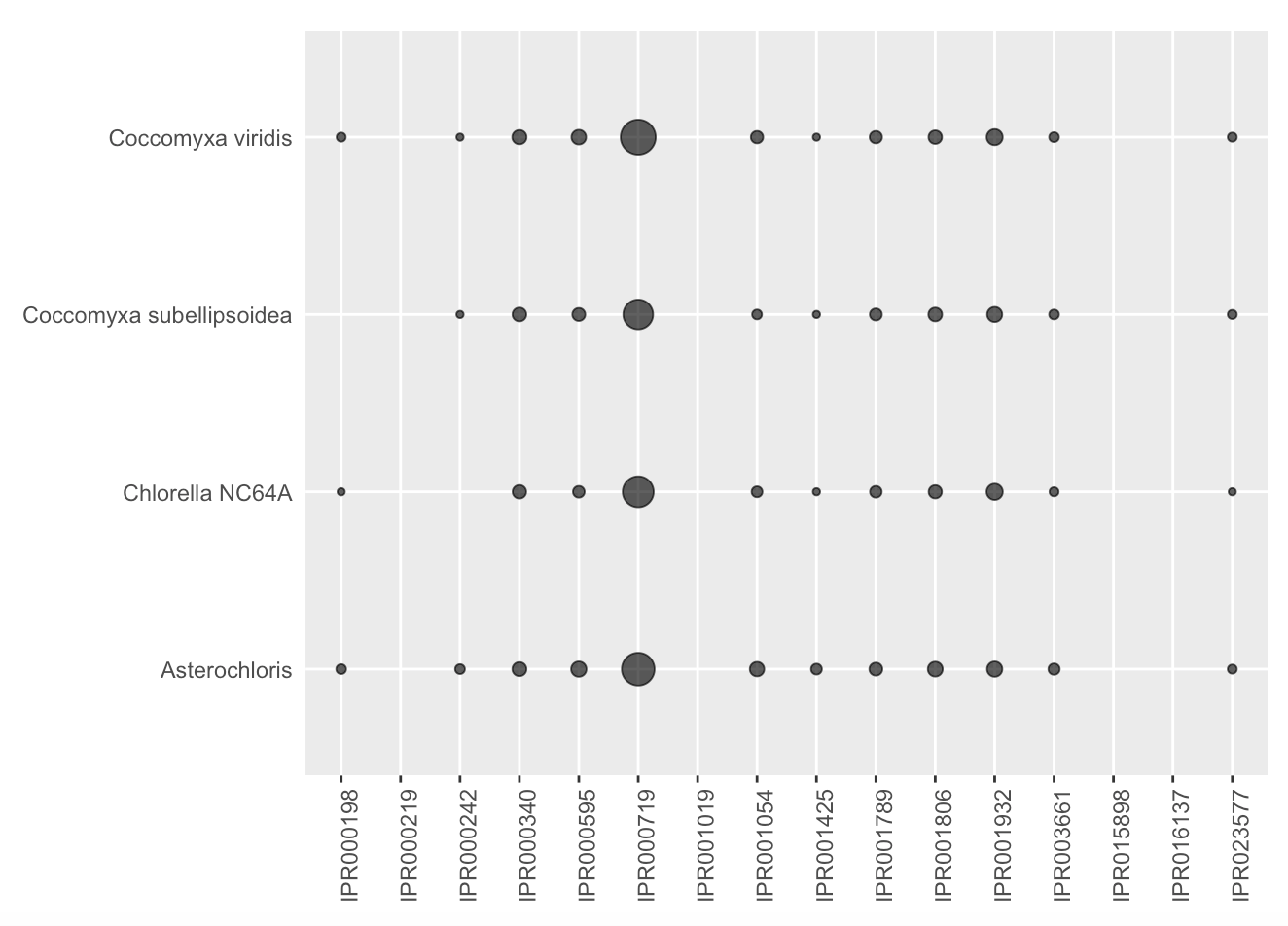


Fig. S2. Presence of InterProScan families associated with signal transductions in four Trebouxiophyceae genomes. Here we show only functional families highlighted by Armaleo et al. [30] as potentially relevant to lichenization in green algae. The size of the bubbles represent the number of genes assigned to each family in a given genome.
